# 冷冻诱导相分离结合液相色谱-串联质谱快速测定人血清中全氟和多氟烷基化合物

**DOI:** 10.3724/SP.J.1123.2024.11028

**Published:** 2025-07-08

**Authors:** Jiandi WANG, Yiwei WANG, Jiaxin WU, Zhixiong SHI

**Affiliations:** 1.首都医科大学公共卫生学院，北京 100069; 1. School of Public Health，Capital Medical University，Beijing 100069，China; 2.北京市顺义区妇幼保健院，北京儿童医院顺义妇儿医院，北京 101300; 2. Shunyi Maternal and Children’s Hospital of Beijing Children’s Hospital，Beijing 101300，China

**Keywords:** 全氟和多氟烷基化合物, 冷冻诱导, 血清, 同位素稀释, 液相色谱-串联质谱, per- and polyfluoroalkyl substances （PFASs）, cold-induced, serum, isotope dilution, liquid chromatography-tandem mass spectrometry （LC-MS/MS）

## Abstract

全氟和多氟烷基化合物（per- and polyfluoroalkyl substances， PFASs）的广泛应用带来了显著的环境污染与人群健康风险。血清测定是开展PFASs的人群内暴露评估及人群流行病学研究的基础，因此亟需建立可快速准确测定大规模人群血清中PFASs 的分析技术。本研究建立了可快速测定血清中31种PFASs的方法。血清中加入稳定性同位素内标后采用乙腈萃取，通过集净化与富集为一体的冷冻诱导相分离处理后，采用液相色谱-电喷雾电离-串联质谱在选择反应监测模式下采集质谱数据，内标法定量。对仪器分析参数和冷冻诱导相分离条件进行了优化。确定以乙腈为萃取溶剂，冷冻诱导相分离时溶液中乙腈体积分数为35%，-20 ℃下冷冻1 h为最佳样品前处理条件。在优化后的条件下，PFASs在0.5~20 ng/mL范围内线性良好，检出限为0.01~25 pg/mL，定量限为0.03~83 pg/mL；在5和25 ng/mL加标水平下，PFASs的平均回收率为60.5%~129.6%，相对标准偏差≤22.8%；在5 pg/mL定量限加标水平下，PFASs的平均回收率为61.6%~199.1%，RSD≤29.4%。采用所建立的方法测定了北京市顺义区50份孕妇血清样本，其中PFASs总和含量中值与均值分别为21.8和22.9 ng/mL，含量范围为0.456~73.9 ng/mL。有24种PFASs检出率在30%以上，其中9种检出率大于80%。高检出率表明PFASs的人群暴露普遍存在，同时胎儿可通过母-婴传递暴露于PFASs。此外传统PFASs和新型替代品检出率和污染水平均较高，人群暴露和潜在健康风险不容忽视。本研究所建立的方法操作简单，有机溶剂和耗材消耗极少，灵敏度和稳定性良好，适用于大规模人群监测与环境流行病学研究。

## Abstract (en)

Per- and polyfluoroalkyl substances （PFASs） are a large group of synthetic chemicals that have been widely used in various industrial and commercial products owing to their unique physicochemical properties. However， accumulating evidence suggests that PFASs are persistent， transmissive over long distances， bioaccumulative， and toxic； consequently， their adverse effects on ecosystems and humans is of widespread concern. Serum is the most commonly used human matrix for assessing internal exposure to environmental pollutants， and several analytical methods have been developed to measure PFASs in sera. Current methods are generally fast， convenient， and robust； however， their pretreatment steps require large amounts of organic solvents and materials， such as solid-phase extraction cartridges and/or sorbents. In this study， a novel and low-cost analytical method based on cold-induced phase separation （CIPS） strategy was developed for the simultaneous determination of 31 legacy and emerging PFASs in serum. The core mechanism and distinctive feature of CIPS involves cooling an acetonitrile-water （ACN-water） mixture at a low temperature to produce two clear-cut layers： one with a high ACN proportion （the ACN layer） and an aqueous layer （water layer）. Certain chemicals are significantly enriched in the ACN layer during cooling； at the same time， impurities， especially water-soluble impurities， remain in the aqueous layer. CIPS only requires the temperature to be varied， and no external impurities are introduced during pretreatment， which dramatically reduces material costs and avoids new impurities from intervening. Our method involves the following procedure： serum was drawn accurately （0.2 mL） into a 1.5 mL Eppendorf （EP） tube， 2 ng of each isotopically labeled internal standard was added， the mixture is vortexed， and 350 µL of ACN was added， followed by vortexing and ultrasonic extraction. Subsequently， 450 µL of water is added to adjust the volume proportion of ACN to 35% （the volume percentage of ACN in the total solution）. The protein at the bottom of the tube was collected following centrifugation at 15 000 r/min for 10 min， and the supernatant was transferred to a 1 mL syringe. The syringe was frozen in a -20 ℃ refrigerator for 1 h to obtain the two layers， after which the upper layer （approximately 80–100 μL） containing ACN and the target compounds was finally transferred to a glass vial for instrumental analysis. Liquid chromatography coupled with triple quadrupole mass spectrometry augmented with electrospray ionization （LC-ESI-MS/MS） was used to quantify the PFASs. The analytes were separated using a C18 column， with methanol and 2 mmol/L of ammonium formate-H_2_O used as mobile phases. Linearities， limits of detection （LODs） and， limits of quantification （LOQs）， recoveries， precisions， and matrix effects were determined under the optimal conditions. The LODs and LOQs of PFASs in serum were 0.01–25 and 0.03–83 pg/mL， respectively. Under two spiked levels， namely 5 ng/mL and 25 ng/mL， average recoveries ranged between 60.5% and 129.6%， with relative standard deviations （RSDs） of less than 22.8%. Under 5 pg/mL as LOD spiked level， average recoveries ranged between 61.6% and 199.1%，with RSDs<29.4%. While matrix-effect testing revealed slightly enhanced signals， the use of isotopically labeled internal standards compensated for these effects. Real samples were subsequently analyzed， with 50 human serum samples collected in first trimester of pregnancy women living in the Shunyi District， Beijing. Nine PFASs exhibited high detection frequencies （>80%）， which suggests that PFASs are ubiquitous in the population. The median and mean levels of Σ_31_PFASs （sum of 31 PFASs） in serum were 21.8 and 22.9 ng/mL， respectively， and the range was 0.456-73.9 ng/mL. Both legacy and emerging PFASs were detected at high frequencies and contamination levels， which suggests that they are widely used. In summary， the method developed in this study is fast， sensitive， and solvent- and material-efficient； it is also very linear and highly accurate， and exhibits satisfactory extraction recovery and enrichment factors； hence， it is suitable for surveying large populations as well as for use in environmental epidemiology.

全氟和多氟烷基化合物（per- and polyfluoroalkyl substances，PFASs）是一类含有高能碳氟（C-F）共价键的小分子化合物的统称，由于性质稳定并具有特殊的疏水和疏油性，被广泛应用于工业生产和各类民用产品中，但PFASs的生产、使用和产品废弃过程也带来了环境污染以及生态健康风险^［1］^。体内外实验已表明，PFASs具有遗传、生殖、神经和发育毒性，以及内分泌干扰作用和疑似致癌性^［2］^。PFASs同时具有难降解，以及在环境中呈现持久性和生物富集性等特点，是国际公认的“永久性化合物（forever chemicals）”^［1，3］^。全氟辛酸（pefluorooctanic acid， PFOA）和全氟辛烷磺酸 （perfluorooctane sulfonate， PFOS）等部分PFASs已被列入《关于持久性有机污染物的斯德哥尔摩公约》中，但由于难以找到合适的替代品，已使用多年的一些传统全氟化合物仍在使用。同时，近年来作为替代品的一些多氟类化合物的使用量在迅速上升，由此带来的人群暴露与健康风险亟待明确。血清是评估环境污染物人群内暴露特征并开展环境流行病学研究的主要基质^［4］^，现有研究已开发出若干种测定血清中PFASs的方法。由于挥发性较弱，PFASs的仪器分析以液相色谱-串联质谱法为主^［5，6］^。在前处理方面，目前多采用固相萃取法^［7，8］^或基质固相分散萃取法^［9］^进行样本处理与净化，也有研究采用离子对液液萃取法^［10］^。现有前处理方法具有快速简便、稳定可靠且处理通量高等优点，但缺点是需消耗大量昂贵试剂和耗材。冷冻诱导相分离技术（cold-induced phase separation，CIPS）是近期发展起来的一种快速、简便、低成本的前处理技术^［11，12］^，其核心原理是使用乙腈萃取待测化合物后，加入适量水混合成适当比例的乙腈-水混合物，随后低温冷冻促进相分离形成乙腈层和水层，在相分离过程中特定化合物会显著富集至乙腈层，同时杂质尤其是水溶性杂质会保留在水层中。相分离后乙腈层可直接取出进行液相色谱-质谱分析。CIPS的显著优势是仅需极少量有机溶剂，且无需使用固相萃取柱或吸附剂等昂贵耗材，大大降低了前处理成本，同时最大限度地避免了前处理过程中外来杂质的引入。本研究结合CIPS和液相色谱-串联质谱法，建立了血清中31种PFASs的同时前处理与定量方法，并用于孕妇血清样本的测定，为开展PFASs的大规模人群流行学研究提供技术支持。

## 1 实验部分

### 1.1 仪器、试剂与材料

UltiMate 3000型高效液相色谱仪、TSQ Altis Quantum Ultra三重四极杆质谱仪（美国Thermo Fisher Scientific公司）；5430 R型高速冷冻离心机（德国Eppendorf公司）。

31种PFASs标准溶液和9种同位素内标标准溶液均购自天津阿尔塔科技有限公司，纯度高于98%（表1），用甲醇稀释配制成合适浓度的混合标准工作液及内标混合工作液。实验用水为超纯水（Milli-Q纯水仪，美国默克公司）；甲酸铵、甲醇和乙腈均为色谱纯（美国Thermo Fisher Scientific公司）；用于方法验证实验的胎牛血清购自上海ExCell Bio公司。实际血清样本来自北京市顺义区妇幼保健院所建立的“顺义妇幼健康之约”队列，所有血清捐献者已被明确告知队列研究目的并签署知情同意书。本研究已获得顺义区妇幼保健院医学伦理委员会审查通过（审编号2024-021-2）。

**表1:** 31种PFASs和9种内标的保留时间和质谱参数

No.	Compound	Chinese name	RT/min	Precursor ion （*m/z*）	Product ion （*m/z*）	CE/eV	RF/V	IS
1	perfluorobutyric acid （PFBA）	全氟丁酸	3.77	213.1	169.0^*^	10	30	^13^C_4_-PFBA
					180.8	10		
					182.8	17		
2	perfluorobutanesulfonate （PFBS）	全氟丁烷磺酸盐	6.02	298.9	80.0^*^	33	103	^18^O_2_-PFHxS
					99.0	30		
					218.9	23		
3	sodium 1*H*，1*H*，2*H*，2*H*-perfluorohexane sulfonate（4∶2） （4∶2FTS）	1*H*，1*H*，2*H*，2*H*-全氟己烷磺酸钠	6.65	327.0	80.4	28	77	^13^C_4_-PFBA
					286.9	25		
					306.9^*^	20		
4	perfluorohexanoic acid （PFHxA）	全氟己酸	6.78	312.9	119.1	21	30	^13^C_2_-PFHxA
					269.0^*^	10		
					295.2	17		
5	perfluoropentanesulfonic acid （PFPeS）	全氟膦酸	6.9	349.1	80.0^*^	35	105	^18^O_2_-PFHxS
					99.0	32		
					119.0	33		
6	hexafluoropropylene oxide dimer acid （HFPO-DA）	六氟环氧丙烷二聚酸	6.93	285.0	119.0	29	62	^13^C_2_-PFHxA
					169.0^*^	10		
					185.0	19		
7	perfluorobutane sulfonamide （FBSA）	全氟丁烷磺酰胺	6.99	298.0	78.0^*^	25	70	^13^C_4_-PFOA
					119.0	19		
					218.8	19		
8	perfluoroheptanoic acid （PHHpA）	全氟庚酸	7.46	362.9	169.0	17	32	^18^O_2_-PFHxS
					281.1	17		
					319.0^*^	10		
9	sodium dodecafluoro-3*H*-4，8-dioxanonanoate （NaDONA）	十二氟-3*H*-4，8-二氧六烯酸钠	7.48	377.0	85.0	29	38	^13^C_4_-PFOA
					251.0^*^	10		
					358.9	10		
10	dodecafluoro-3*H*-4，8-dioxanonane sodium salt （DONA）	十二氟-3*H*-4，8-二氧杂壬酸钠	7.53	376.7	250.9^*^	10	40	^13^C_4_-PFOA
11	perfluorohexanesulfonate （PFHxS）	全氟己烷磺酸	7.53	399.1	80.0^*^	23	112	^18^O_2_-PFHxS
					99.0	37		
					319.0	35		
12	perfluorohexyl ethyl vinyl ether sulfonic acid （6∶2FTS）	全氟己基乙烯基醚磺酸	7.92	427.0	292.9	34	94	^13^C_4_-PFOA
					386.9	29		
					406.8^*^	23		
13	perfluorooctanoic acid （PFOA）	全氟辛酸	7.99	413.1	169.0	17	39	^13^C_4_-PFOA
					219.0	16		
					369.0^*^	10		
14	perfluoroheptanesulfonic acid （PFHpS）	全氟庚烷磺酸	8.01	448.9	80.0^*^	38	127	^13^C_4_-PFOA
					99.0	38		
					169.0	33		
15	perfluorohexane sulfonamide （FHxSA）	全氟己烷磺酰胺	8.29	398.0	78.0^*^	28	91	^13^C_4_-PFOS
					169.0	27		
					377.9	21		
16	perfluorononanoic acid （PFNA）	全氟壬酸	8.41	462.8	219.0	16	42	^13^C_5_-PFNA
					269.0	17		
					419.0^*^	10		
17	perfluorooctanesulfonate （PFOS）	全氟辛烷磺酸	8.41	498.9	80.0^*^	41	109	^13^C_4_-PFOS
					99.0	40		
					230.0	40		
18	6∶2 chlorinated polyfluorinated ether sulfonate （6/2F-53B）	6∶2氯代多氟烷基醚磺酸盐	8.62	531.0	351.0^*^	26	97	^13^C_2_-PFDA
19	8∶2 fluorotelomer thioether amido sulfonate （8∶2FTS）	8∶2氟代烷基硫醚酰胺磺酸盐	8.72	527.0	392.9	38	110	^13^C_2_-PFDA
					486.8	33		
					506.8^*^	27		
20	perfluorodecanoic acid （PFDA）	全氟癸酸	8.73	513.0	219.0	18	45	^13^C_2_-PFDA
					269.0	18		
					469.0^*^	10		
21	perfluorononanoic acid （PFNS）	全氟壬烷磺酸	8.76	549.1	80.0^*^	43	125	^13^C_2_-PFDA
					98.9	42		
					119.0	43		
22	*N*-methyl perfluorooctanesulfonamide （*N*-MeFOSAA）	*N*-甲基全氟辛烷磺酰胺	8.89	570.0	418.9^*^	20	86	^13^C_5_-PFNA
					482.7	16		
					511.9	21		
23	*N*-ethyl perfluorooctane sulfonamido acetic acid （*N*-EtFOSAA）	*N*-乙基全氟辛烷磺酰氨基乙酸	9.03	584.0	418.9^*^	20	75	^13^C_4_-PFOA
					482.9	16		
					525.9	20		
24	perfluoroundecanoic acid （PFUdA）	全氟十一酸	9.06	563.1	219.0	18	50	^13^C_2_-PFUdA
					269.0	18		
					519.0^*^	10		
25	perfluorodecanesulfonate （PFDS）	全氟癸烷磺酸	9.06	598.9	80.0^*^	47	175	^13^C_2_-PFUdA
					99.0	45		
26	perfluorooctane sulfonamide （FOSA）	全氟辛烷磺酰胺	9.08	498.0	78.0^*^	30	105	^18^O_2_-PFHxS
					429.7	13		
					477.9	25		
27	11-chloroperfluoro-3-oxaundecanesulfonic acid （11Cl-PF3OUDS）	11-氯二十氟-3-氧杂十一烷-1-磺酸	9.16	631.0	35.2	46	93	^13^C_2_-PFDoA
					450.9^*^	29		
					468.9	23		
28	8∶2 chlorinated polyfluorinated ether sulfonic acid （8/2F-53B）	8∶2氯代多氟烷基醚磺酸盐	9.20	630.9	450.9^*^	28	122	^13^C_4_-PFOS
					549.0	16		
29	perfluorododecanoic acid （PFDoA）	全氟十二酸	9.32	613.1	319.0	18	56	^13^C_2_-PFDoA
					530.9	15		
					569.0^*^	10		
30	perfluorotridecanoic acid （PFTrDA）	全氟十三酸	9.56	663.1	318.9	19	59	^13^C_2_-PFDoA
					369.0	19		
					619.0^*^	10		
31	perfluorotetradecanoic acid （PFTeDA）	全氟十四酸	9.76	713.0	318.9	20	62	^13^C_2_-PFDoA
					630.9	17		
					669.0^*^	10		
32	perfluoro-*n*-（1，2，3，4-^13^C_4_）butanoic acid （^13^C_4_-PFBA）	^13^C_4_-全氟丁酸	3.77	217.0	19.2	55	30	
					157.1	17		
					172.1^*^	10		
33	perfluoro-*n-*（1，2-^13^C_2_） hexanoic acid （^13^C_2_-PFHxA）	^13^C_2_-全氟己酸	6.71	315.0	91.0	14	35	
					119.4	22		
					270.0^*^	10		
34	sodiumperfluro-1-hexane（^18^O_2_）sulfonate （^18^O_2_-PFHxS）	^18^O_2_-全氟己烷磺酸	7.53	403.0	84.0^*^	36	158	
					103.0	35		
					168.9	31		
35	perfluoro-*n*-（1，2，3，4-^13^C_4_）octanoic acid （^13^C_4_-PFOA）	^13^C_4_-全氟辛酸	7.94	417.0	172.1	18	37	
					318.8	11		
					372.0^*^	10		
36	perfluoro-*n*-（1，2，3，4，5-^13^C_5_）nonanoic acid （^13^C_5_-PFNA）	^13^C_5_-全氟壬酸	8.36	468.0	172.1	19	41	
					218.8	17		
					422.8^*^	10		
37	perfluoro-*n*-（1，2，3，4-^13^C_4_）octanesulfonate （^13^C_4_-PFOS）	^13^C_4_-全氟辛烷磺酸	8.36	503.0	80.0^*^	41	100	
					99.0	41		
					111.0	27		
38	perfluoro-*n*-（1，2-^13^C_2_）decanoic acid （^13^C_2_-PFDA）	^13^C_2_-全氟癸酸	8.7	515.1	219.2	18	51	
					269.4	18		
					470.0^*^	12		
39	perfluoro-*n*-（1，2-^13^C_2_）undecanoic acid （^13^C_2_-PFUdA）	^13^C_2_-全氟十一酸	9.02	565.3	269.4	18	42	
					319.3	18		
					520.1^*^	10		
40	perfluoro-*n*-（1，2-^13^C_2_）dodecanoic acid （^13^C_2_-PFDoA）	^13^C_2_-全氟十二酸	9.32	615.0	319.3	18	53	
					369.3	18		
					570.0^*^	10		

RT： retention time； CE： collision energy； RF： radio frequency； IS： internal standard； * quantitative ion.

### 1.2 样品前处理

取含内标50 pg/μL的内标混合工作液40 μL于1.5 mL 离心管中，氮气吹干溶液中的甲醇后加入200 μL血清样品并涡旋振荡1 min；加入350 μL乙腈，再次涡旋振荡1 min，随后超声萃取20 min；加入纯水450 μL，涡旋30 s后在15 000 r/min下离心10 min；取上清液转移至1 mL一次性注射器（7.2 cm×0.5 cm）中，于直立状态下置于-20 ℃冰箱中冷冻1 h后，在保持冷冻分层状态下迅速使用长型玻璃巴斯德吸管吸取上层液体（80~100 μL），转移至带玻璃内插管的进样小瓶中待上机检测。前处理过程中避免使用聚四氟乙烯材质的器皿以控制外源性污染。

### 1.3 液相色谱-质谱条件

Shim-pack GISS-HP C18色谱柱（150 mm×2.1 mm，3 μm，日本岛津公司）；流动相：A相为2 mmol/L甲酸铵水溶液，B相为甲醇，流速0.4 mL/min。柱温箱温度35 ℃，进样体积5 μL。梯度洗脱程序：0~0.5 min，20%B；0.5~9 min，20%B~100%B；9~12 min，100%B；12~15 min，20%B。

离子源：电喷雾电离源（ESI），负离子模式；电喷雾电压2 500 V；离子传输线温度325 ℃；气化温度350 ℃。以氮气作为鞘气和辅助气，流速分别设置为50和10 Arb；数据采集模式：选择反应监测模式（SRM）。31种PFASs的质谱分析参数及每种PFASs对应内标如表1所示。

### 1.4 萃取回收率和富集系数计算方法

采用萃取回收率（Rec.）和富集系数（enrichment factor，EF）评价CIPS萃取并富集PFASs的效率。萃取回收率定义为相分离后乙腈层中待测物质量与原始样本液中的质量之比。富集系数定义为乙腈层中待测物浓度（*C*_u_）与原始样本溶液中的浓度（*C*_i_）之比。计算公式如下：

Rec.=(*C*_u_×*V*_u_)/(*C*_i_×*V*_i_) (1)

EF=*C*_u_/*C*_i _ (2)

式中， *V*_u_和*V*_i_分别为相分离后乙腈层的体积（依据注射器刻度测量）和原始样本液体积。

## 2 结果与讨论

### 2.1 仪器分析条件的优化

使用各PFASs标准溶液进行全扫描，确定各目标物的保留时间以及母离子和产物离子，每个化合物确定一对定量离子对和至少两对定性离子对。由于部分PFASs尤其是新型多氟化合物尚未有商品化同位素内标，需与其他化合物共用内标。依据待测物与内标需物理化学性质近似，且保留时间与质谱响应相近的原则，结合加标回收结果优选出各待测物最适宜的内标物。对碰撞能量、离子源温度、传输线温度、梯度程序等条件进行优化，使31种PFASs最大程度上实现分离且达到最佳响应，获得的最优质谱参数见表1，混合标准溶液的总离子流色谱图如图1所示。

**图1:**
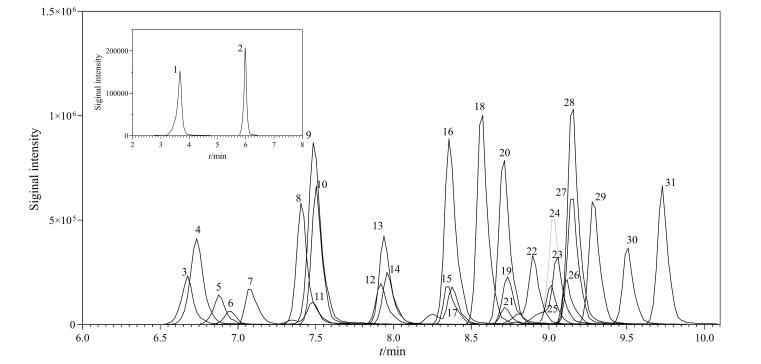
31种PFASs标准溶液（5 ng/mL）的总离子流色谱图

### 2.2 样品前处理条件的优化

#### 2.2.1 CIPS前处理步骤简介

CIPS前处理大致分为3个步骤。首先使用有机溶剂进行液液或固液萃取提取待测物。乙腈可有效提取各类不同极性的有机物，是最常用的提取溶剂，本实验中乙腈在萃取PFASs的同时还能有效沉淀并通过离心除去血清中的蛋白质。第二步是向萃取液中加入适量水以调节乙腈在溶液中的比例（由于血清基本成分为水，因此实验中0.2 mL血清也视为0.2 mL水）。第三步是在适宜温度和时间下冷冻诱导相分离。第三步是CIPS的独特特征，其核心原理是合适比例的乙腈-水混合物在低温下可相分离形成乙腈层和水层，在相分离过程中特定化合物会显著富集至乙腈层，同时杂质尤其是水溶性杂质保留在水层中。相分离后乙腈层可直接取出进行液相色谱-质谱分析。

#### 2.2.2 相分离时溶液中乙腈比例的优化

在冷冻诱导相分离过程中，溶液中乙腈占总体积的比例是影响待测物回收率和富集系数的主要因素。通常随着乙腈比例的降低，乙腈层体积也会缩小，同时富集系数会提高，直至乙腈比例下降至冷冻诱导相分离无法发生为止^［13］^。本研究以胎牛血清作为模拟物，通过对胎牛血清加标样品（10 ng/mL）的测定探索最优乙腈比例。在维持血清（0.2 mL）-乙腈-水混合液总体积为1 mL的前提下，通过改变所加入乙腈和水的体积，探索不同乙腈比例下PFASs的富集系数和萃取回收率。实验中观察到当乙腈比例（乙腈体积占溶液总体积的比例）低于30%时相分离难以发生，而当乙腈比例过高（大于70%）时冷冻后乙腈层体积过大，不利于待测物的富集，因此较佳的乙腈比例应控制为30%~70%。随后探索了乙腈比例从35%上升至70%时各待测物的富集系数和萃取回收率（见图2）。所有PFASs的富集系数随着乙腈比例的上升均呈现显著的下降趋势（图2a），尤其是乙腈比例从35%上升到40%时，富集系数下降幅度达50%以上，说明当乙腈比例控制在35%时PFASs能更有效地富集至乙腈层，而随着乙腈比例的上升，乙腈层体积增大导致其中待测物浓度显著降低，不利于待测物的仪器分析。从萃取回收率来看（图2b），随着乙腈比例的上升，大部分PFASs（PFBA、PFBS、PFHxA、PFPeS、PFHxS、PFOA、PFOS、8/2F-53B、PFNA、PFUdA、PFDS、PFDoA、PFDA等）的回收率均在100%左右且无剧烈变动，说明这些化合物及其内标物在相分离过程中无论乙腈比例如何变化，均能稳定地富集到乙腈层中。另有部分化合物在低乙腈比例（35%~40%）下具有较好的回收率，但随着乙腈比例的增高有变差的趋势，包括FBSA、6/2F-53B、*N*-MeFOSAA、*N*-EtFOSAA、PFTeDA、11Cl-PF3OUDS等。综合考虑，为满足大部分PFASs的准确测定，本研究决定控制冷冻诱导相分离时的乙腈比例为35%，即溶液中乙腈为350 μL，水为650 μL。

**图2:**
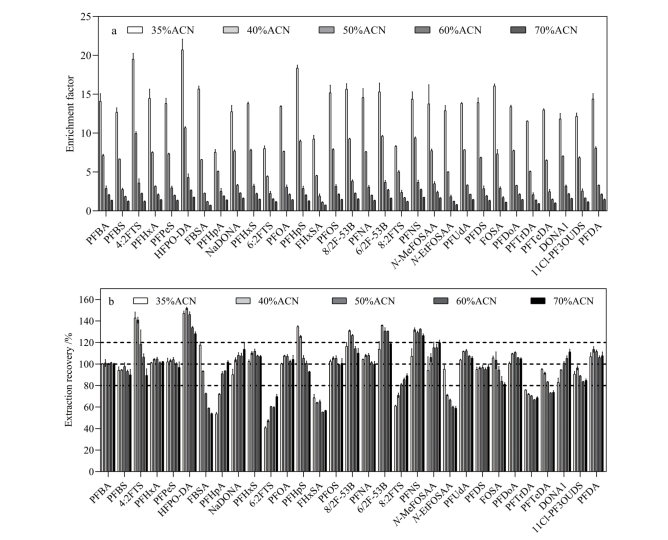
不同乙腈比例下PFASs的（a）富集系数和（b）萃取回收率（*n*=5）

#### 2.2.3 冷冻诱导温度和时间的优化

除了乙腈比例，冷冻诱导温度和时间也会影响相分离。通常低温可促进相分离，当乙腈比例控制在35%时，发现在超低温（-80 ℃）条件下，溶液在几分钟内便会迅速冻结，导致乙腈和水无法分离。在-40 ℃条件下冷冻40 min后形成清晰稳定的乙腈层和水层。在-20 ℃冰箱中也能形成清晰稳定的乙腈层和水层，但冷冻时间需在1 h以上，时间过短会导致相分离不完全并影响待测物富集和水相中蛋白质沉淀。但如果冷冻时间过久，容易在乙腈层中形成絮状冰晶影响后续乙腈层的吸取转移。考虑到-20 ℃冰箱更为常用，建议在溶液总体积为1 mL时，冷冻诱导以-20 ℃冷冻1 h为宜。

### 2.3 方法验证

#### 2.3.1 标准曲线与检出限

配制系列标准溶液，标准溶液中PFASs的质量浓度分别为0.5、1、5、10和20 ng/mL，内标均为5 ng/mL。以各PFASs与相应内标的峰面积之比作为纵坐标（*y*），PFASs与相应内标的浓度之比作为横坐标（*x*），得到各目标物的标准曲线和相关系数（*R* ^2^）。结果显示，各PFASs在0.5~20 ng/mL范围内线性良好，相关系数均大于0.99（表2）。以3倍信噪比计算方法的检出限（LOD），10倍信噪比计算方法的定量限（LOQ），PFASs的LOD为0.01~25 pg/mL，LOQ为0.03~83 pg/mL（表2）。

**表2:** 31种PFASs的回归方程、相关系数、检出限、定量限和加标回收率（*n*=5）

Analyte	Regression equation	*R* ^2^	LOD/ （pg/mL）	LOQ/ （pg/mL）	5 pg/mL		5 ng/mL		25 ng/mL	
					Recovery/%	RSD/%	Recovery/%	RSD/%	Recovery/%	RSD/%
PFBA	*y*=0.8248*x*+0.0211	0.9996	25	83	67.8	27.1	96.6	13.4	85.4	1.8
PFBS	*y*=1.6518*x*‒0.0060	0.9995	0.019	0.06	61.7	10.7	109.6	11.5	98.9	0.7
4∶2FTS	*y*=0.4176*x*+0.0071	0.9998	0.01	0.03	100.1	16.5	91.8	3.6	88.5	1.4
PFHxA	*y*=1.1525*x*‒0.0107	0.9996	6.3	21	140.3	7.2	109.1	9.6	80.8	1.5
PFPeS	*y*=1.0098*x*+0.0083	0.9999	0.21	0.7	125.7	7.6	91.7	17.4	78.2	0.3
HFPO-DA	*y*=0.0957*x*+0.0014	0.9981	0.39	1.30	91.1	23.1	106.9	18.2	99.8	2.2
FBSA	*y*=0.6353*x*‒0.0123	0.9998	0.21	0.7	72.8	29.4	105.6	13.1	97.2	1.7
PFHpA	*y*=4.3409*x*‒0.0443	0.9994	2.6	8.7	103.2	6.7	85.5	16.8	90.9	4.8
NaDONA	*y*=1.5939*x*‒0.0098	0.9999	0.83	2.77	102.6	3.2	79.1	13.1	90.1	5.5
PFHxS	*y*=0.8123*x*+0.0063	0.9999	1.3	4.33	139.3	21.6	95.3	14.5	88.7	0.6
6∶2FTS	*y*=0.2976*x*+0.0038	0.9999	0.01	0.03	120.7	25.8	119.4	11.9	92.7	5.3
PFOA	*y*=0.6552*x*+0.0035	0.9999	3.3	11	195.4	19.5	92.5	13.6	96.9	4.6
PFHpS	*y*=0.2650*x*+0.0007	0.9999	0.03	0.1	86.2	7.8	90.8	12.9	119.3	1.8
FHxSA	*y*=1.9626*x*‒0.0120	0.9997	0.58	1.93	62.3	12.9	94.5	9.4	88.8	0.9
PFOS	*y*=1.2570*x*+0.0090	0.9997	0.97	3.23	199.1	5.4	115.1	7.0	78.6	0.9
8/2F-53B	*y*=11.2170*x*‒0.0721	0.9997	0.19	0.63	163.8	10.7	92.2	9.7	85.3	7.5
PFNA	*y*=1.2058*x*‒0.0163	0.9993	2.2	7.33	150.4	16.4	96.1	11.6	79.3	0.9
6/2F-53B	*y*=2.9025*x*‒0.0283	0.9999	0.57	1.9	163.8	10.7	91.0	15.5	86.5	4.9
8∶2FTS	*y*=0.3688*x*+0.0007	0.9998	0.095	0.32	110.3	9.8	118.3	11.6	96.3	0.5
PFNS	*y*=0.1777*x*+0.0020	0.9998	0.01	0.03	150.4	16.4	96.8	14.4	76.2	0.1
*N*-MeFOSAA	*y*=0.3315*x*‒0.0027	0.9994	0.88	2.93	106.5	5.9	109.7	11.7	129.6	5.9
*N*-EtFOSAA	*y*=0.2330*x*‒0.0009	0.9997	0.23	0.77	143.9	16.9	95.8	22.2	106.1	2.4
PFUdA	*y*=0.7296*x*+0.0019	0.9999	0.6	2	177.7	7.3	94.5	22.8	82.8	1.6
PFDS	*y*=0.2290*x*+0.0002	0.9999	0.02	0.07	61.6	6.6	90.5	9.4	79.7	0.6
FOSA	*y*=0.5112*x*‒0.0085	0.9999	0.28	0.93	102.9	22.9	98.4	12.9	92.2	1.2
PFDoA	*y*=0.6711*x*‒0.0032	0.9995	1.3	4.33	96.0	1.2	107.5	11.5	85.3	1.7
PFTrDA	*y*=0.6481*x*‒0.0021	0.9999	0.53	1.77	95.8	9.9	92.4	12.1	81.5	3.2
PFDA	*y*=0.8668*x*‒0.0050	0.9999	0.97	3.23	158.7	14.8	97.0	12.7	81.4	1.0
PFTeDA	*y*=0.9004*x*‒0.0207	0.9998	0.81	2.7	94.9	1.1	91.8	12.3	86.2	4.7
DONA1	*y*=1.5079*x*‒0.0097	0.9999	1.7	5.67	98.8	2.8	60.5	5.9	72.5	3.9
11Cl-PF3OUDS	*y*=1.5204*x*‒0.0009	0.9999	0.12	0.4	69.2	8.1	80.6	16.8	74.5	0.5

*y*： peak area ratio of the target to internal standard； *x*： mass concentration ratio of the target to internal standard.

#### 2.3.2 加标回收试验

以胎牛血清作为加标基质，设定定量限（5 pg/mL，本研究中PFASs的平均定量限）、低（5 ng/mL）和高（25 ng/mL）3个加标水平开展加标回收试验（表2）。在定量限加标回收试验中，各化合物的平均加标回收率为61.6%~199.1%，RSD≤29.4%。部分化合物，例如PFOA和PFOS，在极低的加标量下受血清和试剂耗材中存在的背景污染干扰严重，导致加标回收率偏高。而某些新型PFASs，例如PFDS、8/2F-53B和*N*-EtFOSAA，由于没有对应的同位素内标，导致加标回收率偏高或偏低。在低和高加标回收试验中，各化合物的平均加标回收率分别为60.5%~119.6%（RSD≤22.8%）和72.5%~129.6%（RSD≤7.5%）。加标回收实验结果表明本方法具有较好的回收率和精密度。

### 2.4 基质效应

虽然前处理过程中可将生物基质中的蛋白质和脂肪等大部分杂质通过沉淀方式除去^［14］^，但残存的一些杂质仍有可能在质谱分析中抑制或增强待测物响应。为评估前处理后残存杂质对目标物定量的影响，本研究用建立的前处理方法对血清样品进行前处理，获得空白基质溶液。将标准工作液分别添加到空白基质溶液和甲醇中以达到相同的加标水平，制备基质匹配溶液和试剂标准溶液并进行LC-MS/MS测定。以基质匹配溶液和试剂标准溶液中各待测物峰面积比评估基质效应。该比值<0.5或>1.5表示基质效应强烈，体现为显著抑制或增强^［15］^。本实验计算了待测物在5 ng/mL和50 ng/mL两个加标水平下的基质效应，结果显示在血清样中各PFASs均呈现出较弱的基质增强效应（比值范围1.1~1.5）。但随着待测物浓度的降低，基质效应出现增强趋势。通常，基质增强效应由杂质中某些可增强离子化效率的成分引起。在基质效应实验中，不同加标水平下，待测物浓度降低的同时溶液中杂质含量并未改变，因此低浓度加标液中的待测物受增强离子化效率的影响更严重，导致低浓度加标样本出现基质效应更强的趋势。虽然仅观察到较弱的基质效应，但在PFASs的检测中稳定性同位素内标的使用依然必不可少。

### 2.5 实际样品测定

已有动物实验表明孕期PFASs暴露可显著影响胎体生长发育^［16］^，因此亟需明确大规模人群孕期PFASs暴露水平，以便开展人群流行病学研究。本研究使用已建立的方法测定了孕妇孕早期（<孕13周）血清样本50份（表3）。有24种PFASs在血清中检出率在30%以上，其中9种检出率在80%以上。高检出率表明PFASs的人群暴露普遍存在，同时胎儿可通过母-婴传递暴露于PFASs。血清中PFASs总和含量中值与均值分别为21.8和22.9 ng/mL，含量范围为0.456~73.9 ng/mL。血清含量水平较高的依然是PFHxS、PFPeS、PFOA和PFOS等传统PFASs，但部分新型替代品如6/2F-53B和6∶2FTS也具有很高的检出率和含量，表明目前市场上传统和新型PFASs均存在大量使用的现象，人群暴露和潜在健康风险不容忽视。

**表3:** 孕妇孕早期血清中PFASs的含量

Compound	DF/%	Contents/（ng/mL）		
		Median	Mean	Max
PFBA	46	-	0.146	1.27
PFBS	90	0.115	0.114	0.315
4∶2FTS	72	0.247	0.843	5.17
PFHxA	76	0.008	0.089	0.523
PFPeS	88	3.1	3.84	14.0
HFPO-DA	36	-	0.058	0.31
FBSA	4	-	0.001	0.002
PFHpA	38	-	0.014	0.168
NaDONA	48	-	0.003	0.029
PFHxS	90	5.63	5.35	15.7
6∶2FTS	64	1.25	2.99	57.7
PFOA	68	1.54	2.18	12.3
PFHpS	88	0.09	0.09	0.237
FHxSA	8	-	0.001	0.008
PFOS	90	3.73	4.28	12.6
8/2F-53B	26	-	0.002	0.003
PFNA	90	0.491	0.52	1.53
6/2F-53B	90	1.09	1.30	7.71
8∶2FTS	12	-	0.049	1.21
PFNS	88	0.031	0.030	0.091
*N*-MeFOSAA	32	-	0.004	0.033
*N*-EtFOSAA	6	-	0.001	0.031
PFUdA	78	0.272	0.329	1.32
PFDS	90	0.026	0.029	0.126
FOSA	14	-	0.001	0.015
PFDoA	64	0.02	0.032	0.15
PFTrDA	32	-	0.026	0.151
PFDA	66	0.289	0.524	3.17
PFTeDA	36	-	0.005	0.059
11Cl-PF3OUDS	8	-	0.003	0.064
DONA	34	-	0.005	0.047
∑方正汇总行_31_PFASs		21.8	22.9	73.9

DF： detecting frequency. -： no median value due to low detecting frequency （<50%）.

## 3 结论

本研究基于冷冻诱导相分离技术结合液相色谱-串联质谱法建立了血清中多种PFASs同时前处理与定量的分析技术。该方法操作简单，可节省大量有机溶剂及耗材，并具有较好的准确度和精密度，适用于大规模人群的内暴露监测。
